# Immunosuppressive Therapies in Pulmonary Sarcoidosis: A Practical, Evidence-Based Review

**DOI:** 10.3390/jcm14196828

**Published:** 2025-09-26

**Authors:** Zehra Dhanani, Rohit Gupta

**Affiliations:** 1 Thoracic Medicine and Surgery, Temple University Hospital, Philadelphia, PA 19140, USA; 2 Thoracic Medicine and Surgery, Lewis Katz School of Medicine, Temple University, Philadelphia, PA 19140, USA

**Keywords:** steroid sparing agents, inflammation, patient centered approach

## Abstract

Sarcoidosis is a chronic inflammatory disease of unknown etiology that can involve virtually any organ, with pulmonary involvement seen in over 90% of cases. Although many patients experience spontaneous remission, approximately 10–30% develop progressive pulmonary disease, which may lead to fibrocystic changes, respiratory failure, and death. Oral glucocorticoids remain the cornerstone of treatment for symptomatic patients with pulmonary infiltrates and abnormal pulmonary function tests, with typical starting doses ranging from 20 to 40 mg/day followed by a slow taper over 6–18 months based on clinical and radiographic response. However, prolonged glucocorticoid therapy is associated with significant toxicity, and many patients require additional immunosuppressive agents for disease control or steroid-sparing purposes. Antimetabolites such as methotrexate, azathioprine, mycophenolate mofetil, and leflunomide are commonly used second-line therapies. For refractory disease, particularly in those with metabolically active lesions on FDG-PET, anti-tumor necrosis factor (TNF) agents like infliximab may be effective but carry risks of serious adverse effects. In select cases, newer strategies—including RCI, rituximab, JAKi or investigational regimens—are being explored. Management must also account for non-inflammatory complications such as sarcoidosis-associated pulmonary hypertension and bronchiectasis, which can mimic disease progression and require distinct therapeutic approaches. Given the heterogeneity of sarcoidosis and lack of robust clinical trial data, a stepwise and individualized approach to immunosuppression remains essential in optimizing outcomes while minimizing treatment-related harm.

## 1. Introduction

**Figure 1:**
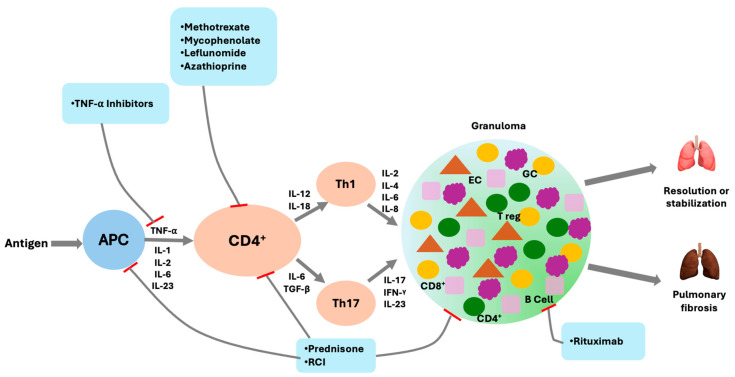
Mechanistic schematic of sarcoidosis immunopathogenesis and therapeutic targets. In this schematic the antigen-presenting cell (APC) is a macrophage that activates CD4^+^ T cells, driving T-helper 1 (Th1) and T-helper 17 (Th17) responses and granuloma formation with epithelioid cells (ECs), multinucleated giant cells (GCs), CD4^+^ and CD8^+^ T cells, B cells, and regulatory T cells (Tregs). Antimetabolites (methotrexate, mycophenolate, leflunomide, azathioprine) inhibit T-cell proliferation. Glucocorticoids and repository corticotropin injection (RCI) broadly suppress T-cell activation and cytokine production. Tumor necrosis factor alpha (TNF-α) inhibitors neutralize TNF-α central to granuloma maintenance. Rituximab depletes B cells. The clinical trajectory may culminate in resolution or stabilization or progress to pulmonary fibrosis.

Sarcoidosis is a multisystem granulomatous disease of unknown etiology characterized by the formation of non-caseating granulomas in affected organs. The lungs and intrathoracic lymph nodes are involved in over 90% of cases, making pulmonary sarcoidosis the most common and clinically significant manifestation [1,2]. Many patients remain asymptomatic or have nonprogressive diseases that may spontaneously remit; however, in a minority, persistent inflammation can progress, leading to irreversible lung damage and pulmonary fibrosis [3,4]. Figure 1 illustrates both the immunopathogenic mechanisms driving granuloma formation and the potential clinical trajectories, emphasizing that ongoing inflammation in some patients culminates in fibrosis while others experience stabilization or resolution.

**Figure 2:**
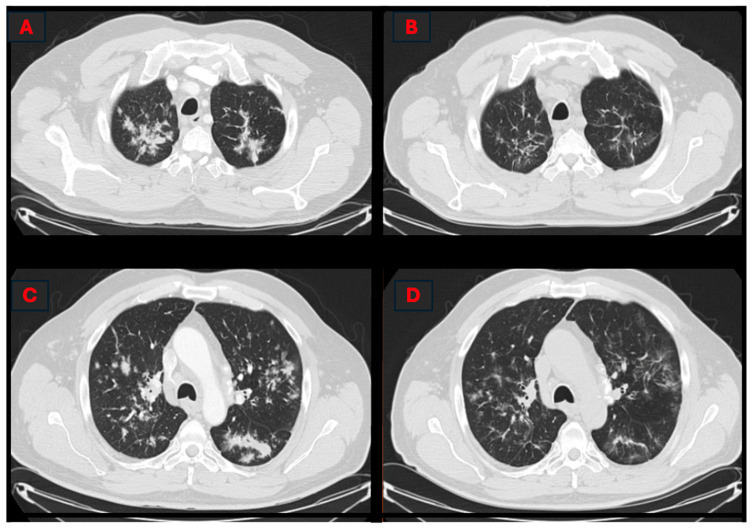
Axial CT images of the chest before (**A**,**C**) and after (**B**,**D**) treatment with immunosuppressive therapy, demonstrating marked reduction in parenchymal opacities and nodularity, consistent with radiologic improvement in pulmonary sarcoidosis.

Initiating immunosuppressive therapy in pulmonary sarcoidosis requires a nuanced, individualized approach guided by symptom severity, disease trajectory, and risk of progression. Key considerations include the severity of respiratory symptoms, impact on quality of life, risk of disease progression, and potential for irreversible organ damage. Many patients are asymptomatic or have nonprogressive diseases and may experience spontaneous remission; in such cases, observation without treatment is often appropriate. However, for patients with more severe or progressive disease, immunosuppressive therapy can reduce granulomatous inflammation, improve symptoms, stabilize or enhance pulmonary function, limit radiographic progression, and prevent long-term complications. Radiologic improvement following immunosuppressive treatment is illustrated in Figure 2. Once therapy is initiated, treatment decisions should be guided by symptom burden, lung function trends, imaging findings, and drug tolerability, along with minimizing long-term glucocorticoid exposure [5,6,7,8,9]. The patient’s unique characteristics, comorbidities, and symptom profile should remain central to treatment decision-making.

This review provides a comprehensive overview of available immunosuppressive therapies, beginning with corticosteroids and progressing through commonly used steroid-sparing agents, biologics, and other emerging options. Table 1 summarizes key immunosuppressive agents and relevant considerations.

We performed a targeted narrative review using PubMed/MEDLINE, Embase, and the Cochrane Library from January 2000 through September 2025; ClinicalTrials.gov was screened for ongoing or recently completed trials. Search terms combined ‘sarcoidosis’ or ‘pulmonary sarcoidosis’ with ‘glucocorticoids,’ ‘methotrexate,’ ‘azathioprine,’ ‘leflunomide,’ ‘mycophenolate,’ ‘infliximab,’ ‘adalimumab,’ ‘rituximab,’ ‘repository corticotropin,’ ‘JAK inhibitor,’ ‘mTOR,’ and related terms. We prioritized randomized trials, large observational cohorts, guideline or consensus statements, and studies reporting lung function, imaging, or patient-reported outcomes; English-language adult studies were included, and landmark earlier studies were retained when foundational.

## 2. First Line Agents

### 2.1. Glucocorticoids

Glucocorticoids remain the cornerstone of initial therapy for sarcoidosis and are considered first-line treatment for patients with symptomatic pulmonary disease. Their mechanism of action is both immunosuppressive and anti-inflammatory, targeting multiple steps in granuloma biology. By binding to cytosolic glucocorticoid receptors and modulating gene transcription, they suppress Th1/Th17-driven inflammation, inhibit proinflammatory cytokine production (e.g., TNF-α, IL-2, IFN-γ), and reduce macrophage activation and granuloma formation [12]. Randomized trials and systematic reviews have demonstrated modest but consistent short-term benefits, including improvements in chest radiographic findings, symptom scores, and spirometry over 3 to 24 months—particularly in patients with parenchymal involvement [13,14]. However, there is limited evidence that glucocorticoids improve long-term lung function metrics such as forced vital capacity (FVC) or Diffusing Capacity of the Lungs for Carbon Monoxide (DLCO), and data remain insufficient to determine whether they alter the long-term course of disease [13,14]. As such, current recommendations support their use in patients with moderate to severe or progressive symptoms or radiographic changes, while emphasizing the need for individualized treatment decisions based on progression risk and potential toxicity [5,6]. Importantly, European Respiratory Society (ERS) guidelines highlight that glucocorticoids are not recommended in patients who are asymptomatic, have stable lung function, or demonstrate isolated stage I disease, where observation without therapy is appropriate [5].

Longer-term data provide further support for early glucocorticoid use. Some trials have shown that early systemic corticosteroids followed by inhaled therapy may improve 5-year lung function and reduce persistent radiographic abnormalities in patients with stage II–III disease [15]. Other studies have reported modest but statistically significant improvements in symptoms and lung function with prolonged corticosteroid therapy compared to as needed use [16]. However, follow-up data suggest that these benefits may not persist after treatment is discontinued, highlighting the potential for spontaneous improvement in some patients and the need for careful selection of those most likely to benefit [17]. Additional evidence indicates no clear dose–response relationship between cumulative prednisone exposure and improvement in FVC, reinforcing the importance of minimizing exposure whenever possible [18].

Clinical guidelines consistently recommend initiating oral prednisone at 20–40 mg daily, followed by a gradual taper to the lowest effective dose over 3 to 6 months [5,6,9]. In practice, treatment duration varies widely—ranging from few months to two years—depending on disease severity, response, and chronicity [5,13]. The British Thoracic Society (BTS) clinical statement emphasizes the importance of shared decision-making, with careful discussion of both potential benefits and the considerable risks of long-term toxicity [9]. For patients with relapsing or refractory disease, early introduction of steroid-sparing agents is advised to minimize cumulative glucocorticoid exposure [5,6,9].

Despite their clinical effectiveness, glucocorticoids carry significant toxicity, especially with prolonged or high-dose use. Higher cumulative exposure—particularly >500 mg of prednisone in a year—has been associated with significantly worse health-related quality of life, especially in domains such as fatigue, daily activities, and overall satisfaction, even after adjusting for disease severity [19]. In fact, propensity analyses suggest that fatigue, functional limitation, and reduced daily activity levels are disproportionately worse among glucocorticoid-treated patients compared to untreated individuals, even when lung function is stable [19]. Glucocorticoid-treated patients also face elevated risks of weight gain, hypertension, metabolic syndrome, and cardiovascular disease. Even after adjustment for demographic and clinical factors, the risk of adverse events remains more than double compared to untreated individuals [20]. In addition, there is a strong association with increased incidence of type 2 diabetes, particularly within the first two years of treatment [21]. These findings underscore the need to use the lowest effective dose for the shortest duration necessary, with early transition to alternative agents when ongoing immunosuppression is required.

### 2.2. Methotrexate

Methotrexate (MTX) is the most widely studied and commonly used steroid-sparing agent in the management of pulmonary sarcoidosis. At low weekly doses, it increases extracellular adenosine and suppresses T-cell-mediated inflammation, dampening granuloma formation [22]. Historically reserved for patients with glucocorticoid toxicity, dependence, or relapsing disease, MTX has recently emerged as a potential first-line therapy, supported by a high-quality comparative trial [10].

Early studies established methotrexate’s role in reducing glucocorticoid exposure while maintaining disease control. In a double-blind, randomized trial, the addition of methotrexate to a standardized prednisone taper in patients with new-onset sarcoidosis significantly reduced cumulative glucocorticoid use over six months. Patients receiving methotrexate required less prednisone and experienced fewer steroid-associated side effects, with no increase in adverse events compared to placebo. These findings confirmed the delayed-onset steroid-sparing effect of methotrexate [23].

Subsequent observational studies supported these results, showing that nearly 80% of patients treated with methotrexate demonstrated radiographic and clinical improvement, regardless of baseline characteristics or dose. Importantly, more recent real-world data have shown that methotrexate not only improves radiographic and physiologic outcomes but also leads to meaningful improvements in patient-reported outcomes, including fatigue and overall quality of life. Adverse events were mostly mild and gastrointestinal in nature, with low rates of drug discontinuation, even in the absence of folic acid supplementation [24].

Comparative data have also suggested that methotrexate may offer advantages over glucocorticoids in certain long-term outcomes. In one head-to-head trial, methotrexate monotherapy was associated with a lower relapse rate and less treatment resistance than methylprednisolone, despite similar efficacy and side effect profiles. Increasing the weekly dose from 10 mg to 15 mg appeared to accelerate clinical improvement without increasing toxicity, supporting a dose–response relationship within a safe therapeutic range [25].

Based on accumulating evidence, methotrexate is widely endorsed as a second-line agent for patients unable to taper prednisone below 10 mg/day or those experiencing glucocorticoid-related toxicity. It is typically initiated at 10–15 mg once weekly and titrated up to 20–25 mg as tolerated, with folic acid supplementation to reduce adverse effects. Subcutaneous administration is preferred in patients with gastrointestinal intolerance. The World Association of Sarcoidosis and Other Granulomatous Disorders (WASOG) multinational recommendations specifically endorse regular laboratory monitoring with CBC and LFTs, along with folic acid supplementation, as standard of care when prescribing methotrexate in sarcoidosis [26]. Regular laboratory monitoring is essential due to the potential for hepatotoxicity, cytopenias, and, rarely, pulmonary toxicity, although this concern has been frequently challenged, and observational data do not support an increased risk of methotrexate accelerating or worsening underlying interstitial lung disease [7,8,26,27,28,29].

The paradigm shifted further with the landmark PREDMETH trial, a multicenter, randomized, open-label noninferiority study that compared MTX and prednisone as initial therapy in treatment-naïve pulmonary sarcoidosis with moderate to severe symptoms, impaired lung function, and parenchymal abnormalities. At 24 weeks, MTX was noninferior to prednisone in improving FVC, with results numerically favoring prednisone (adjusted between-group difference, −1.17 percentage points; 95% CI, −4.27 to 1.93), and falling well within the prespecified noninferiority margin. While prednisone was associated with more rapid early improvement in FVC, the degree of benefit at 24 weeks was similar in both groups. Importantly, the adverse effect profiles differed: prednisone led to more weight gain, insomnia, and increased appetite, while MTX caused more nausea, fatigue, and abnormal liver tests. Both agents led to comparable improvements in quality of life and fatigue scores. These findings not only validate methotrexate’s role as a steroid-sparing agent but also support its use as a viable first-line option, particularly for patients at high risk for steroid toxicity or those who strongly prefer to avoid glucocorticoids. The PREDMETH trial marks a pivotal moment in sarcoidosis management and may help inform shared decision-making around initial therapy selection [10].

## 3. Second Line Agents

While methotrexate is now supported as a potential first-line alternative to glucocorticoids in selected treatment-naïve patients, it remains the preferred second-line agent for those requiring a steroid-sparing strategy after initial prednisone therapy. Its role and supporting evidence are discussed in detail in the above section.

### 3.1. Azathioprine

Azathioprine (AZA) is a purine analog and prodrug of 6-mercaptopurine that is metabolized to 6-thioguanine nucleotides, which inhibit de novo purine synthesis and impair proliferation of activated T and B lymphocytes. It has long been used as a second-line immunosuppressive agent in pulmonary sarcoidosis, particularly in patients requiring prolonged glucocorticoid therapy or those experiencing steroid-related toxicity. While less extensively studied than methotrexate, AZA remains a viable steroid-sparing option, though its use is tempered by concerns regarding infectious complications and modest overall efficacy.

Initial studies suggested that azathioprine could provide clinical benefit in selected patients with chronic or relapsing sarcoidosis [30,31]. In one early retrospective cohort, the combination of AZA and prednisolone was associated with significant symptomatic relief and improvements in radiographic, physiological, and serological parameters, without serious adverse effects. Most patients achieved clinical remission, reinforcing the potential of AZA as a steroid-sparing agent in long-term disease management [31]. However, one study reported more limited benefits, particularly in patients with fibrotic disease or refractory sarcoidosis. In this cohort, only a minority of patients experienced durable improvement in lung function or radiographic findings, with the majority showing transient or no response. Nevertheless, the safety profile was favorable, with no major toxicities reported [32].

Comparative data provide additional insight into the role of azathioprine. A large international retrospective cohort study evaluated the efficacy of AZA versus methotrexate in pulmonary sarcoidosis. Both agents were found to be similarly effective in reducing prednisone dose—achieving a mean decrease of 6.3 mg/year—and in improving lung function indices, including FEV1, VC, and DLCO. Approximately 70% of patients completing one year of therapy on either agent were able to reduce daily prednisone by at least 10 mg. However, azathioprine was associated with a significantly higher incidence of infections compared to methotrexate (34.6% vs. 18.1%), particularly upper respiratory tract infections, although other adverse effects were comparable. These findings support the utility of AZA as a steroid-sparing agent while favoring methotrexate when infection risk is a key consideration [30].

Current clinical guidelines recommend azathioprine as a second-line option for pulmonary sarcoidosis in patients unable to taper prednisone below 10 mg/day or who develop glucocorticoid-related side effects. The typical dose is 2 mg/kg/day, often administered alongside a reduced glucocorticoid dose. Routine monitoring for cytopenias and infection is essential, especially during the early phases of therapy. Despite its role in current practice, AZA’s place in the treatment algorithm remains based largely on retrospective data and expert consensus.

### 3.2. Leflunomide

Leflunomide is another second-line immunosuppressive agent used in sarcoidosis, though it is less well studied than methotrexate or azathioprine. It acts by inhibiting dihydroorotate dehydrogenase, a key enzyme in de novo pyrimidine synthesis, thereby limiting the proliferation of activated lymphocytes and suppressing the immune response [33]. Its role is primarily supported by small, retrospective studies, and no randomized trials have evaluated its efficacy or safety. Despite these limitations, leflunomide is often considered for patients with chronic or refractory disease, especially those who are intolerant to methotrexate or require additional steroid-sparing strategies [5,6,9].

One retrospective review of 34 patients with chronic sarcoidosis, most with pulmonary or ocular involvement, found that leflunomide—used either alone or in combination with methotrexate—was associated with high partial or complete response rates (75% for pulmonary and 82% for ocular disease). Leflunomide was generally well tolerated, with nausea being the most frequent adverse effect leading to discontinuation. No serious toxicities were reported, and the study suggested that leflunomide may have comparable efficacy to methotrexate with a potentially better side effect profile. However, the data were limited by small sample size and lack of standardized dosing or long-term outcomes [34].

A larger retrospective cohort of 76 patients with pulmonary and extrapulmonary sarcoidosis further supported the use of leflunomide in patients who experienced disease progression or intolerance to other immunomodulators. In this study, leflunomide was associated with a modest improvement in pulmonary function (FVC increased by +0.09 ± 0.3 L compared to a decline of −0.1 ± 0.3 L in the prior six months; *p* = 0.01). Extrapulmonary disease also responded well, with 51% achieving a good response and 32% a partial response. Importantly, the median daily prednisone dose decreased from 10 mg to 0 mg over six months (*p* < 0.001), highlighting its steroid-sparing potential. Adverse effects were noted in approximately one-third of patients, most commonly gastrointestinal, and led to treatment discontinuation in 17% [35].

While these data suggest leflunomide can be an effective steroid-sparing agent, its use remains off-label and largely guided by expert consensus. In current practice, leflunomide is typically reserved for selected patients who have failed or cannot tolerate more commonly used second line agents such as MTX.

### 3.3. Mycophenolate Mofetil

Mycophenolate mofetil (MMF) is used as a second-line immunosuppressive agent in sarcoidosis, particularly in patients with intolerance to other steroid-sparing therapies or with multisystem disease. It works by converting to mycophenolic acid, which inhibits inosine monophosphate dehydrogenase, depleting purine nucleotides and impairing proliferation of activated lymphocytes [36]. However, current evidence supporting its use is limited to small retrospective studies, and its role remains less clearly defined compared to methotrexate or azathioprine.

In patients with chronic pulmonary sarcoidosis, MMF has been associated with steroid-sparing effects and disease stability. One retrospective study of ten patients demonstrated a significant reduction in corticosteroid dose over a median of 31 months without severe adverse events [37]. Another small case series found clinical, radiologic, and functional improvement—including significant gains in FEV_1_ and FVC—and reduction in prednisone dose in patients with systemic sarcoidosis, including cardiac, renal, and skin involvement [38].

However, MMF may be less effective in patients who have already failed other immunosuppressive agents. A larger retrospective cohort suggested no additional benefit in this setting, though patients intolerant to prior therapies did show trends toward improved DLCO and reduced steroid burden. These findings suggest that MMF may be most useful as an alternative in patients who cannot tolerate methotrexate or azathioprine, rather than as salvage therapy for refractory disease [39].

Overall, MMF appears to be well tolerated and offers a viable steroid-sparing option for select patients, particularly those with both pulmonary and extrapulmonary sarcoidosis. In clinical practice, it is frequently considered in patients with multi-organ involvement such as renal, cardiac, or neurologic disease. Clinicians are familiar with its use in the solid-organ transplant setting, which provides confidence in dosing, monitoring, and long-term safety. This background has supported its adoption in sarcoidosis, particularly when other second-line agents are not tolerated. Further prospective studies are needed to clarify its optimal role, comparative efficacy, and long-term safety.

## 4. Third Line Agents

### 4.1. TNF-α Inhibitors

Tumor necrosis factor alpha (TNF-α) inhibitors are considered third-line therapy in sarcoidosis and are reserved for patients with progressive, refractory, or organ-threatening disease that persists despite treatment with glucocorticoids and at least one non-biologic immunosuppressive agent. They act by inhibiting TNF-α, a cytokine central to antigen-stimulated, cell-mediated immune responses and the formation of noncaseating granulomas, thereby disrupting the inflammatory cascade driving sarcoid pathology [40]. They are particularly considered in severe pulmonary, cardiac, neurologic, cutaneous or multiorgan sarcoidosis when standard therapies are ineffective or not tolerated [5,6,9].

Infliximab is the most extensively studied TNF-α inhibitor in sarcoidosis. In a randomized controlled trial of patients with chronic pulmonary sarcoidosis, infliximab led to a statistically significant, though modest, improvement in FVC at 24 weeks. Specifically, Baughman et al. reported that patients treated with infliximab experienced a mean increase of 2.5% in percent predicted FVC from baseline to week 24, compared with no change in the placebo group, and this difference was statistically significant [41]. While the clinical relevance of this change remains debated, post hoc analyses suggest patients with more severe disease may derive greater benefit. Further real-world evidence from a multicenter study by Sakkat et al. demonstrated high rates of treatment success across multiple organ systems, including neurologic (100%), cutaneous (91.7%), pulmonary (78.6%), and upper airway (71.5%) disease involvement. Treatment success was defined as improvement or stabilization of organ-specific manifestations. In addition, infliximab enabled a 50% reduction in prednisone dose, highlighting a significant steroid-sparing effect [42]. Observational data show that many patients who initially respond experience disease relapse following discontinuation—often within the first year [43]. Risk of relapse appears higher in patients with elevated baseline inflammatory activity, such as high FDG-PET uptake or elevated soluble IL-2 receptor levels. These findings support the need for careful selection and close monitoring when initiating and tapering infliximab. The typical dosing is 3–5 mg/kg IV at weeks 0, 2, and 6, followed by infusions every 4–8 weeks. Concomitant immunosuppressants such as prednisone are often used to reduce the development of anti-drug antibodies [44].

Adalimumab is considered a reasonable alternative in patients who develop intolerance to or antibody-mediated resistance against infliximab. Small observational studies suggest that adalimumab can stabilize or improve lung function, reduce inflammatory activity on PET imaging, and improve quality of life in patients with refractory disease [45,46]. It has also been associated with reductions in corticosteroid dose and disease biomarkers. The most common adverse event is infection, though overall tolerability has been favorable [47,48]. Adalimumab may also be preferred by some patients who favor a subcutaneous route of administration over intravenous infusions.

Etanercept, by contrast, has not demonstrated efficacy in sarcoidosis and is not recommended [49]. Other biologic agents such as golimumab and ustekinumab have also failed to show benefit in clinical trials and are not currently supported for use [50]. Additionally, a case report describes worsening uveal sarcoidosis with the IL-23 inhibitor guselkumab, so IL-23 blockade in sarcoidosis remains investigational [51].

Overall, TNF-α inhibitors offer a targeted therapeutic option for patients with refractory sarcoidosis. Infliximab remains the most validated agent in this class, with emerging support for adalimumab in select scenarios. Their use should be individualized and reserved for cases where conventional immunosuppression is ineffective or poorly tolerated.

### 4.2. Repository Corticotropin Injection

Repository corticotropin injection (RCI), administered subcutaneously, stimulates endogenous corticosteroid production by binding to melanocortin receptors, leading to increased cortisol synthesis and release. Beyond its steroidogenic effects, RCI has direct immunomodulatory and anti-inflammatory actions via other melanocortin receptor pathways and has demonstrated steroid-sparing potential [11,52]. In a prospective study of patients with chronic pulmonary sarcoidosis, 24 weeks of RCI therapy was associated with significant improvement in DLCO, reduction in PET scan activity as measured by standardized uptake value of pulmonary lesions, and better patient-reported outcomes on the King’s Sarcoidosis Questionnaire, particularly in health status and fatigue domains. FVC percent predicted did not change significantly, but prednisone dose was reduced, underscoring its steroid-sparing effect. Lower doses (40 units twice weekly) are as effective as higher doses (80 units twice weekly) and are better tolerated [11]. More recently, the PULSAR phase 4 randomized, double-blind, placebo-controlled trial evaluated RCI 80 units subcutaneously twice weekly for 24 weeks and was stopped early because of low enrollment during the COVID-19 period. Although underpowered for formal statistical testing, results showed trends favoring RCI over placebo, including greater improvement in a composite Sarcoidosis Treatment Score (mean change 1.4 vs. 0.7 at week 24), more frequent glucocorticoid discontinuation by week 24, and maintenance of benefit in the open-label extension through week 48; no new safety signals were identified [53]. Adverse effects mirror those of corticosteroids, including weight gain, glucose imbalance, hypertension, edema, mood changes, and infection risk. Its use is further limited by substantial cost, often exceeding $100,000–$200,000 annually per patient [11,54,55].

### 4.3. Rituximab

Rituximab, a monoclonal antibody targeting the CD20 antigen expressed on the surface of pre-B and mature B lymphocytes [56], has been evaluated as an experimental option for refractory pulmonary sarcoidosis. In an open-label, prospective pilot study of 10 adults with chronic pulmonary sarcoidosis who had failed or were intolerant to corticosteroids and at least one other immunosuppressive agent, rituximab was administered as two intravenous infusions of 1000 mg on days 1 and 15 [55]. At 24 weeks, FVC improved in 5 of 10 patients, with a median increase of 7.7% predicted (range 2.7–14.2%), while DLCO improved in 4 of 10 patients, with a median increase of 6.7% predicted (range 2.2–10.2%). The remaining patients had stable or declining lung function. Although no complete remissions were observed, several patients achieved partial responses. Rituximab was generally well tolerated; one patient developed pneumonia requiring hospitalization, but no deaths or unexpected serious adverse events occurred [57]. These findings suggest that rituximab may provide clinical benefit in selected patients with refractory disease, though responses are variable and infection risk remains a concern.

### 4.4. Janus Kinase Inhibitors

Janus kinase inhibitors (JAKi) represent an emerging therapeutic option in sarcoidosis, with early evidence drawn from small prospective studies, case series, and a recent systematic review. Mechanistically, JAKi bind to Janus kinases and block activation of STAT proteins, disrupting JAK-STAT-mediated cytokine and growth factor signaling. This reduces proinflammatory cytokine production, modulates immune cell activity, and suppresses granulomatous inflammation in sarcoidosis [58]. In a proof-of-concept trial of corticosteroid-dependent pulmonary sarcoidosis, tofacitinib enabled ≥50% prednisone reduction in most patients without deterioration in symptoms or spirometry, suggesting a steroid-sparing effect [59]. Additional open-label data in cutaneous sarcoidosis demonstrated marked skin responses and improvement in some internal organ involvement, while case reports have described pulmonary benefit in select patients unable to tolerate glucocorticoids [60,61].

## 5. Additional Considerations

Hydroxychloroquine is used selectively in sarcoidosis, most often for cutaneous disease and hypercalcemia, but evidence for pulmonary benefit is limited. A randomized study of chloroquine in advanced pulmonary sarcoidosis showed an initial gain in lung function with induction therapy, then only a slower decline during maintenance, with a smaller annual drop in FEV_1_ and fewer relapses compared with observation [62]. Published data, including retrospective series, do not show improvement in lung function with hydroxychloroquine monotherapy in pulmonary sarcoidosis [63]. Hydroxychloroquine is generally preferred over chloroquine because of lower ocular toxicity, but ophthalmologic screening remains essential [27]. Antimicrobial approaches remain investigational. An open-label single-center study of the CLEAR regimen (levofloxacin, ethambutol, azithromycin, rifampin) reported short-term improvements in FVC, six-minute walk distance, and St. George’s Respiratory Questionnaire scores, although only 8 of 15 patients completed therapy [64]. A subsequent multicenter, double-blind, placebo-controlled phase II trial using levofloxacin, ethambutol, azithromycin, and rifabutin found no benefit in FVC, six-minute walk distance, or quality of life, despite reductions in mycobacterial immune responses [65]. On current evidence, broad-spectrum antimycobacterial therapy should not be used routinely for chronic pulmonary sarcoidosis.

## 6. Future Directions

The treatment landscape for sarcoidosis is evolving, with growing interest in targeted therapies and more personalized approaches to care. As we learn more about the molecular pathways and immune profiles that drive disease, there is hope that we can better match patients to the treatments most likely to help them. Biomarkers such as soluble IL-2 receptor levels, PET scan activity, and gene expression signatures are being studied to guide treatment choices and track disease activity over time. New therapies are also making their way into clinical trials—including JAK inhibitors, anti–IL-6 agents, and Efzofitimod (novel Neuropilin-2 inhibitor) for refractory disease as well as OATD-01 (chitotriosidase inhibitor) being explored for use in early inflammatory disease. In parallel, XTMAB-16, a chimeric anti-tumor necrosis factor alpha monoclonal antibody, has shown activity in ex vivo granuloma models and early clinical pharmacology but remains investigational without phase 2 or 3 efficacy data in sarcoidosis [66]. Interleukin-17 pathway inhibitors (for example, secukinumab, ixekizumab, brodalumab) are also investigational; mechanistic and animal data implicate Th17/IL-17 in granuloma maintenance and fibrotic progression, but there are no supportive clinical trials or guideline endorsements in sarcoidosis at this time [67]. mTOR pathway inhibition is another promising line of investigation. In murine models and human tissue, pharmacologic mTORC1 blockade with sirolimus or everolimus reduces granulomatous inflammation and limits fibrosis [68,69], and a small single-center study in cutaneous sarcoidosis reported clinical and histologic improvement with systemic sirolimus [70], but there are no published clinical trials in pulmonary sarcoidosis, so this approach remains investigational. These and other novel strategies may open new doors for patients with difficult-to-treat or high-risk disease. Still, we need long-term data to understand how these options compare in safety, effectiveness, and durability, and how best to scale back therapy when possible, without losing disease control.

## 7. Conclusions

Immunosuppressive therapy remains a cornerstone in the management of pulmonary sarcoidosis for patients with symptomatic, progressive, or high-risk diseases. While glucocorticoids are effective for short-term control, their long-term toxicity underscores the importance of steroid-sparing agents. Methotrexate is the best-studied and most widely recommended second-line option (and likely an alternative first line agent), with azathioprine, leflunomide, and mycophenolate as reasonable alternatives in select patients. Biologics such as infliximab and adalimumab are appropriate for refractory cases, while agents like RCI, rituximab, and JAKi may offer benefit in specific scenarios. Emerging therapies and biomarkers hold promise for more individualized care. Until further high-quality evidence becomes available, treatment decisions should remain patient-centered, balancing efficacy with long-term safety and quality of life.

## Figures and Tables

**Table 1:** Pharmacologic therapies for pulmonary sarcoidosis: mechanism, typical dosing, key toxicities, and role within current guidance. Recommendations synthesize the European Respiratory Society (ERS 2021) guideline [5], the British Thoracic Society (BTS 2020–2021) clinical statement [9], and Delphi consensus [6]. Evidence notes include the PREDMETH trial for methotrexate [10] and the repository corticotropin injection study showing improvement in diffusing capacity for carbon monoxide (DLCO) and King’s Sarcoidosis Questionnaire (KSQ) health status and fatigue scores, with no change in forced vital capacity (FVC) and a steroid-sparing effect [11]. Abbreviations: IV, intravenous; SC, subcutaneous; BID, twice daily; PRO, patient-reported outcomes; MTX, methotrexate; ACTH, adrenocorticotropic hormone; RCI, repository corticotropin injection; TNF-α, tumor necrosis factor alpha; HTN, hypertension.

Agent	Mechanism of Action	Typical Dose	Toxicities	Guidelines Recommendations **
**First Line**				
**Glucocorticoids**	Broad anti-inflammatory	Prednisone 20–40 mg/day	Short-term: Mood changes, insomnia, appetite increase, fluid retention, hyperglycemia Long-term: Weight gain, HTN, osteoporosis, diabetes, cataracts, infections	First line for symptomatic/progressive disease; avoid in asymptomatic stage I (ERS, BTS)
Methotrexate *	Anti-metabolite, inhibits folate pathways	10–25 mg/week + folic acid	Hepatotoxicity, cytopenias, nausea	Preferred second line; alternative first line in selected cases (ERS, Delphi, PREDMETH)
**Second Line**				
**Azathioprine**	Purine synthesis inhibitor	1.5–2.5 mg/kg/day	Cytopenias, infections, hepatotoxicity	Second line if MTX not tolerated; higher infection risk (ERS, BTS)
**Leflunomide**	Pyrimidine synthesis inhibitor	10–20 mg/day	GI upset, hepatotoxicity, cytopenias	Second line if MTX intolerant; limited evidence (ERS, BTS)
**Mycophenolate mofetil**	Purine synthesis inhibitor	500–1500 mg BID	Infections, cytopenias	Second line, esp. in multi-organ disease (ERS, BTS)
**Third Line**				
**TNF-** **α inhibitors: Infliximab, Adalimumab**	Inhibition of TNF-α mediated inflammation	Infliximab: 3–5 mg/kg IV q4–8 weeks, Adalimumab: 40 mg SC weekly or q2 weeks	Infections, antibody formation	Third line for refractory/organ-threatening disease (ERS, BTS)
**RCI**	Endogenous ACTH analog	40–80 units SC 2x/week	Edema, hyperglycemia	Use for refractory disease; improves DLCO/PRO but FVC unchanged
**Rituximab**	Anti-CD20 monoclonal antibody	1000 mg IV x 2 (2 weeks apart), retreatment based on relapse or at about 6–12 months in responders.	Infections, hypogammaglobulinemia	Rescue in highly refractory disease; small studies only (ERS)

* MTX monitoring: Historically second line. May be used as alternative first line in selected patients per the PREDMETH trial. ** Regulatory status: glucocorticoids (not sarcoidosis-specific); methotrexate, azathioprine, leflunomide, mycophenolate, infliximab, adalimumab, rituximab (off-label for sarcoidosis); RCI is FDA-labeled for symptomatic sarcoidosis.
